# The potential role of NF-kB activation and synthesis of pro-inflammatory mediators in the pathogenesis of allergic contact dermatitis induced by thiuram

**DOI:** 10.1186/2045-7022-5-S1-O22

**Published:** 2015-03-11

**Authors:** Cezary Kowalewski, Dagmara Kurpios-Pieca, Iwona Rahden-Staron, Beata Gajewska, Katarzyna Wozniak

**Affiliations:** 1 Medical University of Warsaw, Warszawa; Poland; 2 Department of Biochemistry, Medical Uniwersity of Warsaw, Warszawa; Poland; 3 Medical University of Warsaw, Department of Biochemistry, Warszawa; Poland; 4 Medical University of Warsaw, Department of Immunodermatology, Warszawa, Poland

## 

Among the various substances known to cause occupational allergic contact dermatitis, additives to rubber, especially thiurams are regarded as the most important class of contact allergens. It has been suggest that in the sensitization phase of contact hypersensitivity hapten application induces strong innate immune mechanisms, causing the release of danger signals as reactive oxygen species (ROS) generation and activation of NF-êB leading to IL-1β and other cytokine release. To investigate whether pro-oxidative properties of thiuram might be involved in immunogenic mechanisms we analyzed of ROS generation, activation of NF-êB, expression of IL-1â in murine macrophages model. Murine macrophages were treated by non-toxic concentrations of thiuram which induced oxidative stress as was demonstrated by increased production of ROS, especially superoxide anion. Laser scanning confocal microscopy revealed translocation of p65 subunit of NF-êB from cytoplasm to nucleus characteristically for activation NF-êB. The consequence of NF-êB activation was the increase of IL-1â production. These results indicate that thiuram induces changes characteristic for inflammation therefore it can be supposed that they participate in the pathogenesis of allergic contact dermatitis induced by thiuram.

